# Role of Glucose Metabolism Reprogramming in the Pathogenesis of Cholangiocarcinoma

**DOI:** 10.3389/fmed.2020.00113

**Published:** 2020-04-03

**Authors:** Kishor Pant, Seth Richard, Estanislao Peixoto, Sergio A Gradilone

**Affiliations:** ^1^The Hormel Institute, University of Minnesota, Austin, MN, United States; ^2^Masonic Cancer Center, University of Minnesota, Minneapolis, MN, United States

**Keywords:** cholangiocarcinoma, metabolic reprogramming, aerobic glycolysis, glucose metabolism, warburg effect

## Abstract

Cholangiocarcinoma (CCA) is one of the most lethal cancers, and its rate of occurrence is increasing annually. The diagnoses of CCA patients remain elusive due to the lack of early symptoms and is misdiagnosed as HCC in a considerable percentage of patients. It is crucial to explore the underlying mechanisms of CCA carcinogenesis and development to find out specific biomarkers for early diagnosis of CCA and new promising therapeutic targets. In recent times, the reprogramming of tumor cells metabolism has been recognized as a hallmark of cancer. The modification from the oxidative phosphorylation metabolic pathway to the glycolysis pathway in CCA meets the demands of cancer cell proliferation and provides a favorable environment for tumor development. The alteration of metabolic programming in cancer cells is complex and may occur via mutations and epigenetic modifications within oncogenes, tumor suppressor genes, signaling pathways, and glycolytic enzymes. Herein we review the altered metabolism in cancer and the signaling pathways involved in this phenomena as they may affect CCA development. Understanding the regulatory pathways of glucose metabolism such as Akt/mTOR, HIF1α, and cMyc in CCA may further develop our knowledge of this devastating disease and may offer relevant information in the exploration of new diagnostic biomarkers and targeted therapeutic approaches for CCA.

## Introduction

Cholangiocarcinoma (CCA) is a primary malignancy that originates from the cholangiocytes lining the biliary tree, also known as bile duct cancer. CCA is classified according to anatomical position, as either intrahepatic (iCCA), arising in the liver, and extrahepatic (eCCA), arising outside the liver. Extrahepatic cholangiocarcinoma can then be further classified as perihilar (pCCA) and distal cholangiocarcinoma (dCCA). The majority of CCA tumors are found in the perihilar and distal region, whereas only 10% are reported as intrahepatic, although responsible for 10–20% of all hepatic tumors (1–3). CCA characteristically manifests late with non-specific symptoms. Due to an insufficient knowledge of risk factors, coupled with inaccurate screenings, dependable early diagnosis has proven problematic. Diagnosed at late stages, CCA is among the most deadly cancers with an ~5% 5-year survival rate in patients (3).

## Epidemiology

The incidence of CCA varies considerably between East-West dichotomy, reflecting variations in genetic and environmental risk factors. The highest incidence of CCA is reported in the north-east part of Thailand with rates of (113/100,000) in men and (50/100,000) in women; which is almost 100 fold more than Europe and North America (1-2/100,000), whereas Australia has the lowest number of CCA incidence (with 0.4/100,000). The prevalence of CCA is increasing worldwide for unknown reasons (4). The on-going overall increase in the rate of CCA makes it imperative to understand the disease's etiology and risk factors.

## Risk Factors and Pathogenesis of CCA

Eighty percent of CCA cases in the western world are periodic and have no identifiable risk factor (4, 5). Smoking, alcohol abuse, obesity, and diabetes have not been consistently linked to higher risk, though a small involvement cannot be ruled out (6). Chronic biliary inflammation is another risk factor that has been generally associated with CCA. For instance, in the western world, primary sclerosing cholangitis (PSC) is associated with 10% of CCA cases (6, 7). Because of the often desmoplastic nature of CCA, it is challenging to discriminate between malignant and the benign characteristic of PSC. Liver cirrhosis of mixed etiology conveys a ten-fold relative risk (7). Uncommon defects of biliary anatomies, such as choledochal cysts (bile duct cyst), Caroli's syndrome (intrahepatic biliary cysts), biliary papillomatosis or adenoma, are linked with a high risk (6–30%) of CCA development. The high frequency of malignant transformation of the cells promotes prophylactic resection (6–8). Inborn or acquired defects of the biliary duct in pancreaticobiliary junction allows pancreatic reflux and subsequently leads to chronic cholangitis and increasing risk of CCA. Hepatolithiasis and chronic intraductal gallstones are also predominantly linked to CCA in Asia, as more than 10% of these patients develop CCA (5, 6).

## Altered Metabolism in Cancer

Transformed metabolism is a common property of most cancer cells, including cholangiocarcinoma (9). The most common and one of the first identified biochemical features of cancer cells is uncharacteristic glucose metabolism. Glucose is known as a primary energy and carbon source for the cells, providing not only energy in form of ATP but also metabolites for several anabolic pathways (8, 9). Glucose transporters help the cells uptake glucose, and when in the cytosol, it is metabolized to pyruvate to yield a small amount of ATP by a multi-step path known as glycolysis. In normal healthy cells, the pyruvate derived from glycolysis is mainly transported into the mitochondrial matrix where the pyruvate dehydrogenase (PDH) enzyme complex oxidizes it into acetyl coenzyme A (CoA). Acetyl CoA further participates in the tricarboxylic acid (TCA) cycle, followed by a high-efficiency ATP generation process known as oxidative phosphorylation (OXPHOS). The full oxidation of one glucose molecule can produce up to 38 molecules of ATP (10).

**Figure 1:**
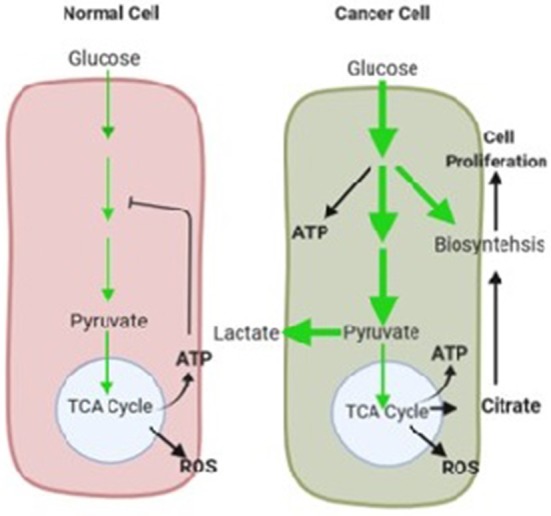
The glucose metabolism in normal healthy and cancer cells under normoxia shown in schematic illustration. The normal cells converted glucose to pyruvate via glycolysis, and maximum amount of pyruvate participates in mitochondrial oxidative process for efficient ATP production. Glucose is largely used for cellular energy requirements. In healthy cells high levels of ATP mitigate glycolysis by feedback inhibition. In cancer cells Glucose uptake increased and thereby glycolysis. A substantial part of glucose is used to biosynthetic pathways to support cell proliferation. Pyruvate is mainly used in lactate production. Oxidative phosphorylation exists, but is separated from augmented glycolysis. The amount of glucose carbon flux is indicated by relative thickness of green arrows. Key mitochondrial metabolism by-products are shown in red and the metabolic effects on cancer are in indicated in bold black arrows.

On the contrary, cancer cells show changes in glucose metabolism. As shown in Figure 1: (i) Comparison between normal healthy cells, cancer cells characteristically show increased glucose uptake and glycolytic rates. Increased consumption of glucose produces more glycolytic intermediate metabolites and a substantial amount of ATP from glycolysis. (ii) Additionally, instead of generating pyruvate, a large fraction of carbon from glucose is moved into multiple biosynthetic pathways. (iii) Finally, in the cytoplasm maximum amount of pyruvate is converted to lactate by the action of lactate dehydrogenase (LDH) and secreted out instead of being oxidized in the mitochondria. This process is aerobic glycolysis as it occurs even in the presence of adequate oxygen to maintain mitochondrial respiration. This phenomenon of cancer cells was first observed by Otto Warburg in 1920s; therefore, it is also referred to as the “Warburg effect” (8, 9). Though cancer cells exhibit various kinds of metabolic profiles, the Warburg effect is a widespread cancer-associated characteristic. Interestingly, higher levels of LDHA have been reported in CCA patients (11, 12).

## Glycolysis Provides Biosynthetic Precursors

The mitochondrial function in cancer cells is mostly unchanged, in opposition to the original Warburg hypothesis. Warburg also witnessed that the rate of mitochondrial respiration in cancer cells remains almost similar to that of normal cells (13). Oxidative metabolism is also present in the majority of cancers and the main source for ATP generation (10, 13). However, the elevated glucose flux in cancer cells predominantly leads to lactate fermentation, and the flow to oxidative metabolism does not change. In summary, augmented glucose intake in cancer cells leads to lactate formation and it is uncoupled from oxidative metabolism in mitochondria. Higher uptake of glucose by cancer cells is also the basis for positron emission tomography (PET) with 18-fluorodeoxyglucose, which particularly accumulate in cancer cells. Due to the prevalence of this phenotype, PET is an effective clinical imaging method to diagnose most cancers and monitor therapeutic approaches (4). This technique has been useful in the clinical diagnosis of CCA in patients (14, 15).

The Warburg effect has been the canter of metabolism research in cancer cells. Genetic and epigenetic alterations in oncogenes and tumor suppressor genes may affect cancer metabolism. Thus, the variations in metabolic activities in the cancer cells are considered as a secondary effect on the cancer development process. Though tumor cell growth requires more production of the basic cellular macromolecules and metabolites for new cell development and it is well known that changes in tumor cell metabolism aid in cell growth by substantially producing biomolecules (16). Glycolytic metabolism of glucose yields several metabolites, which may act as precursors for anabolic pathways including the triacylglycerol and serine biosynthesis pathways for the production of amino acids, nucleotides, and lipids (8, 16). Whereas normal healthy cells utilize glucose almost entirely for energy, cancer cells increase glucose intake mainly to produce a persistent source of glycolytic intermediate products to fulfill the anabolic requirement of proliferating cells. Thus, intermediate product of glycolysis play a very important role as compared to pyruvate. In tumor cells the last steps of glycolysis is slow down, which is controlled by pyruvate kinase (13, 16), permitting glycolytic intermediates build-up for biosynthesis of macromolecules. The transformed metabolism of glucose consequently supports the alteration of glucose into biomass and withstands the rapid growth of cancer cells (9). Modifications in oncogenes and tumor suppressors drive unfavorable cell proliferation, and concurrently maintain cellular metabolism to satisfy the biosynthetic burdens of constant cell proliferation.

## The Regulatory Mechanism in Glucose Metabolism

**Figure 2:**
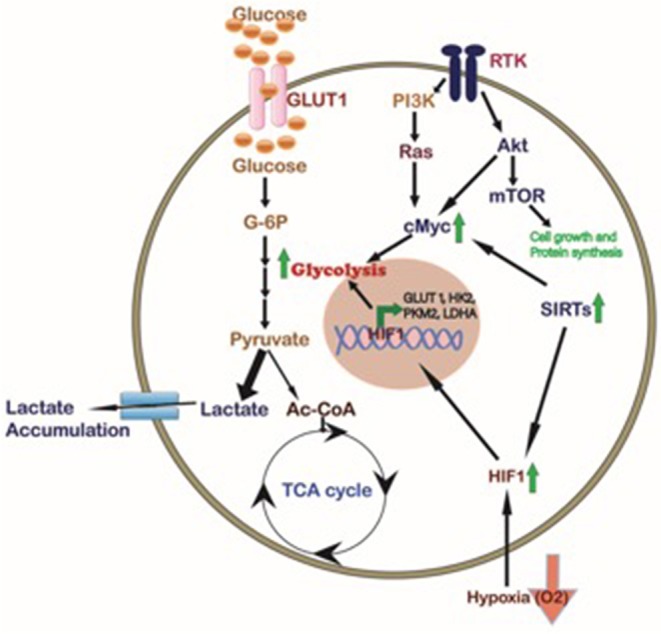
Cellular pathways regulating the cancer cell metabolism. Different signaling pathways affected in tumor cells like Akt/mTOR, KRAS, and SIRTs activate the transcription factor cMyc which in turn, together with HIF1 induced by hypoxia and/or Sirtuins, promotes the expression of glycolytic proteins such as GLUT1, HK2, PKM2, and LDHA to regulate cancer cell metabolism. In CCA activation of HIF1, SIRTs, and cMyc inducing glycolysis has been described (Green Arrows).

Oncogenes are an important set of genes found to be either mutated or over-expressed in cancer cells, which may elicit tumor initiation and continuous cell growth. Healthy cells do not grow independently, but rather go in the cell cycle only after prompted for it via signaling pathways, and growth factors, that may affect the cell morphology and gene expression (13, 17). Given that cell growth depends on many events of metabolic pathways, it is also known that these activities also regulated by the growth factors. The metabolism intermediates regulates metabolic functions that are mostly controlled via the intermediate metabolites on rate-limiting enzymes, providing self-regulatory capability in pathways and gives control at starting points of intersecting pathways (18). A number of these mechanisms in dividing cancer cells help to recognize the influence of signal activities on cell growth and it has shown a range of effects focused on metabolic fluxes (Figure 2).

## The Akt/mTOR Pathway Regulates Aerobic Glycolysis in Cancer Cells

The Akt/mTOR regulatory axis is a highly conserved and commonly expressed system in cells regulated by the growth factors (19). Coupling of growth factors (EGF, IGF, VEGF and cytokines) to cell surface receptors triggers PI3K activation, causing the phosphatidylinositol lipids phosphorylation at the plasma membrane. This process is involved in the activation of downstream pathways, mainly the serine/threonine kinases like Akt/mTOR pathway. Stimulation of the PI3K/Akt/mTOR regulatory axis by the growth factors in cancer cells, augments many of the metabolic processes that support cell growth. Mainly, it allows cells to increases nutrient transporters expression at the cell surface, enabling enhanced nutrient uptake, including, glucose and amino acids (20–22). The Akt/mTOR pathway may affect gene expression and enzyme activity, increase glycolysis and lactate accumulation in the cells, and to prompt aerobic glycolysis (Warburg effect) in either cancer cells or normal cells (22–24). Additionally, initiation of this pathway may enhance the macromolecule biosynthesis. PI3K/Akt encourage expression of lipogenesis genes in various cell types (25), while mTOR is a key controller of protein synthesis in cancer cells (26).

Irrespective of mutation, Akt initiation is among the main signaling events in cancer cell metabolism, for the reason that Akt can initiate aerobic glycolysis and lactate assembly and inhibit degradation of macromolecules in tumor cells including CCA (27). Similarly, CCA cells have been reported to resist the chemotherapy treatment via Akt/mTOR pathway (27).

## HIF1 Modulate the Cancer Cell Metabolism

Hypoxia (decreased oxygen condition) can stimulate increased glucose consumption and lactate production in the cells. This process is regulated by hypoxia-inducible factor 1 (HIF-1), is a transcription factor complex (28, 29). HIF-1 targets genes for glucose transporters, glycolytic enzymes, and LDHA (14, 30). HIF-1 can also expressed in the influence of growth factor signaling, and the Akt/mTOR axis in particular (31–34). During normal oxygen conditions PHD2 induce the posttranslational modification of HIF-1 by prolyl hydroxylation that encourages its interaction with a tumor suppressor called von Hippel-Lindau (VHL), which induces HIF-1 ubiquitination followed by degradation. During hypoxia, prolyl hydroxylation of HIF is withdrawn, resulting in stability and transcriptional activity of the HIF-1 protein complex (35, 36). Cellular stabilization of HIF-1 in normoxia occurs in tumors as an outcome of mutations in the VHL (37, 38).

An interesting study of Xu et al. showed that decreased expression of SIRT3 was correlated with high rate of glycolysis in CCA. Consistently, changes in glucose metabolism were observed in SIRT3 knockout mice. Furthermore, the authors also confirmed that SIRT3 prevents the Warburg effect in CCA cells and a xenograft mouse model by inhibiting the HIF1α/PDK1/PDHA1 pathway. Collectively, this study suggests that SIRT3 shows anti-Warburg effect activity via regulating the HIF1α/PDK1/PDHA1 pathway and may be a potential therapeutic intervention to impair CCA progression (39).

## cMyC

Many previous studies have shown that some glucose metabolism genes are directly regulated by cMyc (40). These genes include Hexokinase 2 (HK2), glucose transporter GLUT1, phosphofructokinase, and enolase 1 (ENO1) (41–43). In addition, cMyc also has been reported to decrease pyruvate levels by stimulating LDHA and PKM2 levels, which can decrease the inhibition of HDAC3 and protect CCA from apoptosis (44). Augmented expression of these genes by cMyc induces the Warburg effect and the capacity of cancer cells to metabolize glucose to pyruvate even in inadequate oxygen availability. Animal models overexpressing cMyc in the liver demonstrated increased glycolytic enzymatic activity and up-regulated lactic acid production. Conversely, rodent fibroblasts cells overexpressing LDHA alone, or cMyc increased the lactate production. This study suggests that LDHA is a downstream target of cMyc, and can induce the Warburg effect (45, 46).

In CCA cells, the SIRT2/cMYC pathway was reported to play a critical role in modulating glucose oxidative metabolism to serine anabolic metabolism. Furthermore, the high level of SIRT2/cMYC pathway not only converts glucose to serine, but also provides antioxidants for oxidative stress resistance in CCA cells. Therefore, the metabolic reprogramming induced by SIRT2/cMYC pathway may provide a new therapeutic target for CCA (47).

## Therapeutic Interventions Related to Reprogrammed Metabolism

Targeting cancer cell metabolism has become a significant emerging field to address effective cancer therapies, including tumor relapse and drug-resistance. To inhibit glucose metabolism in cancer cells, glycolytic enzymes (Pyruvate kinase M2, Hexokinase 1-2, and lactate dehydrogenase) and glucose transporter (GLUT 1-4) have been considered as therapeutic targets (9). GLUT1, found overexpressed in a varity of cancers including CCA (48, 49), helps in rapid uptake of glucose and its expression associates with anaerobic glycolysis in the cells (50). Targeting GLUT1 by WZB117 induced breast cancer cell sensitivity for radiation therapy (51), inhibited cancer cell growth and proliferation in nude mouse (52) and inhibited cancer stem cells self-renewal and tumor development capacity (53). MiR-218, miR-132 and miR-148a have been reported to inhibit cancer cell proliferation via targeting GLUT1 in bladder, prostate and pancreatic cancer respectively (54–56). Therefore, the use of GLUT1 inhibitors in CCA may shed light on the pathogenesis of CCA and may provide novel tools in clinical prognosis and treatment.

Alternative potential target is the adaptive characteristic of cancer cells inside the tumor microenvironment. A cancer cell can transmit its metabolism in heterogeneous environmental conditions, glucose deficiency, hypoxia, and acidic environment; this adaptation to metabolic response by cancer cells plays an essential role in cell metastasis or chemotherapy resistance (16). HIF1α is an important enzyme for metabolic adjustment during hypoxia and is involved in cancer cell survival, angiogenesis, and metastasis (28, 37, 57). CCA patients showed a higher level of Pyruvate dehydrogenase kinase 1 (PDK1) (58), which has been known to control the metabolic shift during hypoxia via regulating the acetyl-coA production to yield energy in the TCA cycle by mitochondria oxidation (59, 60). The mTOR pathway regulates energy homeostasis and engages in cancer cell survival in cellular metabolic stress such as depletion in nutrients and energy (23, 24). The downregulation of mTOR pathway signaling reduces cancer growth and has therapeutic potential for CCA (61–64). CCA cells which need OXPHOS for energy requirements can be targeted by impairing mitochondrial energy metabolism. Targeting the Peroxisome proliferator-activated receptor-γ coactivator (PGC1α) can inhibit the Warburg effect and cause cell death in CCA (65). Inhibition of mitochondrial protein UCP2 can block mitochondrial OXPHOS by the inhibition of mitochondrial function using a UCP2 inhibitor (genipin), which has toxicity against CCA (66). In the same way, TRAP1 a mitochondrial chaperone inhibitor prompts the diminishing of protein folding in mitochondria, also targeting mitochondrial metabolism (67–69). Many studies strongly advocate that targeting OXPHOS in cancer cell mitochondria could be an effective approach to against CCA and to decrease tumor growth and chemotherapy-resistance (70, 71). Therefore, it is crucial to develop the specific therapeutic targets to inhibit mitochondrial OXPHOS only in CCA and for the metabolic alteration of cancer cells with no negative effect in the normal cells that generally use OXPHOS for energy requirements.

## Conclusion

The reprogramming of glucose metabolism in malignant cells is a multi-factor and multi-step route that can be controlled by carcinogenic signaling processes, and by epigenetic modulations. The advancement in cancer metabolism research significantly improved our knowledge of carcinogenesis and gave several potential targets for cancer. The drug that targets glycolytic enzymes or glycolysis pathways revealed several encouraging effects in cancer prevention and therapy (72). The cause and consequences of metabolism reprogramming in CCA is understudied, therefore further efforts to understand how the Warburg effect is regulated are warranted. A better understanding of this topic in CCA might help in the development of novel therapeutic approaches. Nevertheless, the drawback of anti-cancer remedy targeting glycolysis should also be explored further. As several enzymes catalyze this multistep process of cancer metabolism, here is a characteristic compensatory routes in cell metabolism. Thus, drug candidate may not have a prominent effect on cancer metabolism targeting specially only one modulator of glycolysis. In future, The impact of treatments and drug therapies in combination need to be assessed in CCA. In addition, drugs, which can target several pathways like Akt/mTOR and HIF1, are also required to be considered in future studies.

## Author Contributions

KP wrote the first draft of the manuscript. SR, EP, and SG critically revised the manuscript. All authors contributed to manuscript revision, read, and approved the submitted version.

### Conflict of Interest

The authors declare that the research was conducted in the absence of any commercial or financial relationships that could be construed as a potential conflict of interest.
